# Optical Isomers of Atorvastatin, Rosuvastatin and Fluvastatin Enantiospecifically Activate Pregnane X Receptor PXR and Induce CYP2A6, CYP2B6 and CYP3A4 in Human Hepatocytes

**DOI:** 10.1371/journal.pone.0137720

**Published:** 2015-09-14

**Authors:** Martina Korhonova, Aneta Doricakova, Zdenek Dvorak

**Affiliations:** Department of Cell Biology and Genetics, Faculty of Science, Palacky University, Slechtitelu 27, 783 71, Olomouc, Czech Republic; The University of Iowa, UNITED STATES

## Abstract

Atorvastatin, fluvastatin and rosuvastatin are drugs used for treatment of hypercholesterolemia. They cause numerous drug-drug interactions by inhibiting and inducing drug-metabolizing cytochromes P450. These three statins exist in four optical forms, but they are currently used as enantiopure drugs, i.e., only one single enantiomer. There are numerous evidences that efficacy, adverse effects and toxicity of drugs may be enantiospecific. Therefore, we investigated the effects of optical isomers of atorvastatin, fluvastatin and rosuvastatin on the expression of drug-metabolizing P450s in primary human hepatocytes, using western blots and RT-PCR for measurement of proteins and mRNAs, respectively. The activity of P450 transcriptional regulators, including pregnane X receptor (PXR), aryl hydrocarbon receptor (AhR) and glucocorticoid receptor (GR), was assessed by gene reporter assays and EMSA. Transcriptional activity of AhR was not influenced by any statin tested. Basal transcriptional activity of GR was not affected by tested statins, but dexamethasone-inducible activity of GR was dose-dependently and enantioselectively inhibited by fluvastatin. Basal and ligand-inducible transcriptional activity of PXR was dose-dependently influenced by all tested statins, and the potency and efficacy between individual optical isomers varied depending on statin and optical isomer. The expression of CYP1A1 and CYP1A2 in human hepatocytes was not influenced by tested statins. All statins induced CYP2A6, CYP2B6 and CYP3A4, and the effects on CYP2C9 were rather modulatory. The effects varied between statins and enantiomers and induction potency decreased in order: atorvastatin (RR>RS = SR>SS) > fluvastatin (SR>RS = SS>RR) >> rosuvastatin (only RS active). The data presented here might be of toxicological and clinical importance.

## Introduction

Statins are a class of drugs used for the treatment of hypercholesterolemia, a major risk factor for the development of atherosclerotic disease. Statins lower the level of plasma low-density lipoprotein LDL cholesterol by inhibiting 3-hydroxy-3-methylglutaryl-coenzyme A (HMG-CoA) reductase, the enzyme playing a central role in the production of cholesterol in the liver.

**Fig 1:**
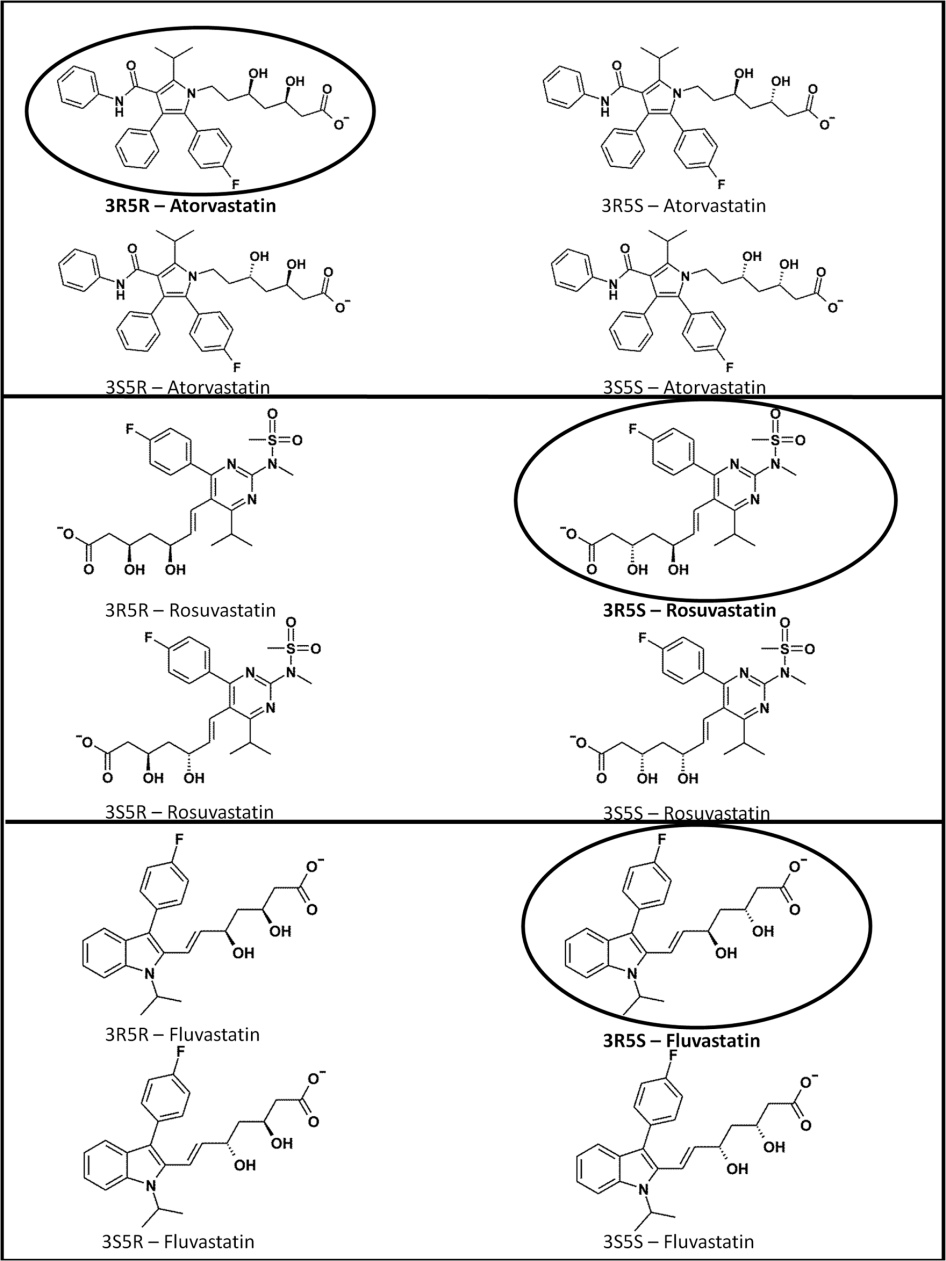
Chemical structures of enantiopure forms of statins. Four individual enantiomers of atorvastatin, rosuvastatin and fluvastatin are shown in the figure. Clinically used enantiopure forms are circled.

Structurally, statins are chiral compounds having two asymmetrical centres in the molecule, enabling formation of four different enantiomers: 3R5R, 3R5S, 3S5R and 3S5S (Fig 1). Individual enantiomers of a drug can qualitatively and quantitatively differ in their biological activities, including their pharmacokinetics, pharmacodynamics, toxicokinetics and toxicodynamics. Notoriously known examples of diastereomers with substantially different biologial activities are R/S-thalidomide, R/S-salbutamol, levo/dextro-methorphan and many others [1]. Therefore, enantiopure drugs have been developed and introduced to the therapy. Regarding the most frequently prescribed statins, following enantiopure formulations are used in the clinics: 3R5R-atorvastatin (Lipitor, Pfizer; since Nov 2011 generic), 3R5S-rosuvastatin (Crestor, AstraZeneca; approved 12^th^ Aug 2003) and 3R5S-fluvastatin (Lescol, Novartis; approved 31^st^ Dec 1993; since 2011 generic).

Statins cause severe adverse effects such as asymptomatic elevation in liver enzyme activity, myopathy and increased risk of diabetes [2]. The risk of adverse effects rises with statins being used simultaneously with other drugs, which may cause drug-drug interactions. Pharmacokinetic of statins is complex. Statins are substrates for multiple membrane transporters, including organic anion transporting polypeptide OATP1B1, breast cancer resistance protein BCRP, and multidrug resistance protein 1 MDR1 [3]. Furthermore, statins undergo substantial microsomal metabolism by the enzyme system of cytochromes P450. Atorvastatin is metabolized mainly by CYP3A4, therefore, inhibitors of CYP3A4 such as protease inhibitor nelfinavir [4] or calcium channel blocker mibefradil [5] affect pharmacokinetics of atorvastatin. Fluvastatin is metabolized primarily by CYP2C9 [6]. Hepatic metabolism of rosuvastatin is predominantly mediated by the CYP2C9 enzyme, with little involvement of CYP3A4 [7]. Therefore, less extent of clinically significant pharmacokinetic drug-drug interactions between rosuvastatin/fluvastatin and other drugs as compared to atorvastatin have been observed [8,9]. Some drug-drug interactions are caused by activation or inhibition of major trasncriptional regulators of drug-metabolizing enzymes, in particular, aryl hydrocarbon receptor (AhR), glucocorticoid receptor (GR) and pregnane X receptor (PXR). Consequently, and induction or down-regulation of drug-metabolizing enzymes may occur. There are numerous reports demonstrating the activation of PXR and induction of PXR-regulated genes by statins [10–14]. Taking in account the presence of two chiral centres in molecules of statins, we tested, whether the effects of statins on the expression of drug-metabolizing cytochromes P450 and the activity of their transcriptional regulators are enantiospecific. Indeed, we have recently demonstrated that several clinically used chiral drugs, including antifungal ketoconazole [15,16], anticoagulant warfarin [17], and proton pump inhibitors omeprazole and lansoprazole [18,19] have enantiospecific effects towards transcriptional regulators (PXR, GR, AhR) of drug-metabolizing enzymes.

The aim of the current paper was to examine stereospecific effects of atorvastatin, fluvastatin and rosuvastatin enantiomers on: (i) the expression of drug-metabolizing cytochromes P450 in primary human hepatocytes; (ii) transcriptional activities of master regulators of drug-metabolizing enzymes, i.e. AhR, GR and PXR receptors, using gene reporter assays. We demonstrate that optical isomers of tested statins activate PXR and induce CYP3A4 in human hepatocytes with enantiospecific pattern. The data presented here might be of toxicological and clinical importance.

## Materials and Methods

### Chemicals

Dimethylsulfoxide (DMSO), rifampicin (RIF), dexamethasone (DEX), mifepristone (RU486), resveratrol, hygromycin B and 3R5R-atorvastatin were purchased from Sigma-Aldrich (Prague, Czech Republic). 2,3,7,8-tetrachlorodibenzo-*p*-dioxin (TCDD) was from Ultra Scientific (Rhode Island, USA). 3R5S- atorvastatin, 3S5R- atorvastatin and 3S5S-atorvastatin were purchased from Toronto Research Chemicals Inc. (Toronto, Canada). 3R5R- fluvastatin, 3R5S- fluvastatin, 3S5R- fluvastatin, 3S5S-fluvastatin, 3R5R- rosuvastatin, 3S5R- rosuvastatin and 3S5S-rosuvastatin were from TLC PharmaChem Inc. (Vaughan, Canada). 3R5S-rosuvastatin was purchased from Santa Cruz Biotechnology Inc. (Heidelberg, Germany). Luciferase lysis buffer and FuGENE® HD Transfection Reagent were from Promega (Madison, California, USA). All other chemicals were of the highest quality commercially available.

### Cell culture

Human Caucasian colon adenocarcinoma cells LS174T (ECACC No. 87060401) and LS180 (ECACC No. 87021202) were purchased from *European Collection of Cell Cultures* (ECACC). Cells were cultured in Dulbecco’s modified Eagle’s medium (DMEM) supplemented with 10% of fetal bovine serum, 100 U/ml streptomycin, 100 μg/ml penicillin, 4 mM L-glutamine, 1% non-essential amino acids, and 1 mM sodium pyruvate. Cells were maintained at 37°C and 5% CO_2_ in a humidified incubator. Stably transfected gene reporter cell lines AZ-AHR and AZ-GR were as described elsewhere [20,21].

Primary human hepatocytes used in this study were isolated from human liver obtained from three multiorgan donors: HH59 (female; 42 years), HH61 (male; 64 years) and HH63 (male; 68 years). The use of liver cells of donors HH59, HH61 and HH63 was approved by “Ethical committee at the Faculty Hospital Olomouc“, and it was in accordance with Transplantation law #285/2002 Sb; "Ethical committee at the Faculty Hospital Olomouc" waived the authors from obtaining consent from the next of kin, regarding human hepatocytes obtained from liver donors HH59, HH61 and HH63. Cells were cultured in serum-free medium. Cultures were maintained at 37°C and 5% CO_2_ in a humidified incubator.

### Gene reporter assay and cytotoxicity assay

A stably transfected gene reporter cell line AZ-AHR, derived from human hepatoma HepG2 cells transfected with a construct containing several AhR binding sites upstream of a luciferase reporter gene, was used for assessment of AhR transcriptional activity [20]. A stably transfected gene reporter cell line AZ-GR, derived from human cervix carcinoma HeLa cells transfected with a construct containing several GR response elements upstream of a luciferase reporter gene, was used for measurement of GR transcriptional activity [21]. A transiently transfected LS180 human colon adenocarcinoma cells were used for assessment of PXR transcriptional activity. A chimera p3A4-luc reporter construct containing the basal promoter (-362/+53) with proximal PXR response element and the distal xenobiotic responsive enhancer module (-7836/-7208) of the CYP3A4 gene 5´-flanking region inserted to pGL3-Basic reporter vector was used. The reporter plasmid was transiently transfected to LS180 cells by lipofection (FuGENE® HD Transfection Reagent). All cell lines were incubated for 24 h with tested compounds and/or vehicle (DMSO; 0.1% v/v), in the presence (antagonist mode) or absence (agonist mode) of 2,3,7,8-tetrachlorodibenzo-p-dioxin (TCDD; 5 nM), rifampicin (RIF; 10 μM) or dexamethasone (DEX; 100 nM). After the treatments, cells were lysed and luciferase activity was measured on Tecan Infinite M200 Pro plate reader (Schoeller Instruments, Prague, Czech Republic). In parallel, cell viability was determined by conventional MTT test (MTT = 3-(4,5-dimethylthiazol-2-yl)-2,5-diphenyltetrazolium bromide).

### mRNA determination and qRT-PCR

Total RNA was isolated using TRI Reagent® (Molecular Research Center, Ohio, USA). cDNA was synthesized from 1000 ng of total RNA using M-MuLV Reverse Transcriptase (New England Biolabs, Ipswich, Massachusetts, USA) at 42°C for 60 min in the presence of random hexamers (New England Biolabs). qRT-PCR was carried out using LightCycler® 480 Probes Master (Prague, Roche Diagnostic Corporation, Czech Republic) on a Light Cycler® 480 II apparatus (Roche Diagnostic Corporation). CYP1A1, CYP1A2, CYP2A6, CYP2B6, CYP2C9, CYP3A4 and GAPDH mRNAs were determined as described previously [22]. Measurements were performed in triplicates. Gene expression was normalized to GAPDH as a housekeeping gene.

### Simple Western blotting by Sally Sue

Total protein extracts were prepared from cells cultured on 6-well plates. Cells were washed twice with ice-cold PBS and scraped into 1 ml of PBS. The suspension was centrifuged (4500 rpm/5 min/4°C) and the pellet was resuspended in 150 μl of ice-cold lysis buffer (150 mM NaCl; 10 mM Tris pH 7.2; 0.1% (w/v) SDS; anti-protease cocktail, 1% (v/v) Triton X-100; anti-phosphatase cocktail, 1% (v/v) sodium deoxycholate; 5 mM EDTA). The mixture was vortexed and incubated for 10 min on ice and then centrifuged (15000 rpm/13 min/4°C). Supernatant was collected and the protein content was determined by the Bradford reagent.

All reagents used for running the *simple western by Sally Sue* ^*TM*^ were obtained from ProteinSimple (San Jose, California) and prepared according to manufacturer’s recommendations (http://www.proteinsimple.com/sally_sue.html). CYP1A1 (goat polyclonal, sc-9828, G-18), CYP1A2 (mouse monoclonal, sc-53614, 3B8C1), CYP2A6 (mouse monoclonal, sc-53615, F16P2D8), CYP2B6 (rabbit polyclonal, sc-67224, H-110), CYP3A4 (mouse monoclonal; sc-53850, HL3) primary antibodies and rabbit anti-goat secondary antibody (sc-2768) were purchased from Santa Cruz Biotechnology Inc. β-actin (mouse monoclonal; 3700S, 8H10D10) primary antibody was from Cell Signalling Technology (Denvers, Massachusetts, USA). CYP2C9 (rabbit polyclonal, AV41809, QC17985) was purchased from Sigma-Aldrich (Prague, Czech Republic). Antibody diluent, goat anti-rabbit secondary antibody, and goat anti-mouse secondary antibody were purchased from ProteinSimple. The capillaries, containing a proprietary UV-activated chemical linked reagent and 384-well plates were obtained from ProteinSimple. All samples and reagents were prepared according to the recommended ProteinSimple manual. Target proteins were identified using primary antibodies and immunoprobed using a horseradish peroxidase-conjugated secondary antibody and chemiluminescent substrate. The resulting chemiluminescent signal was detected and quantified by the Compass Software version 2.6.5.0 (ProteinSimple). For quantitative data analysis, the CYPs signals were normalized to β-actin as a loading control.

### Electrophoretic mobility shift assay EMSA

Electrophoretic mobility shift assay was performed in nuclear fractions from LS174T cells using Nuclear extract kit (Active Motif) according to manufacturer's protocol. Consequently, nuclear fractions were incubated for 2 h at 30°C with DMSO (0.1% v/v), RIF (10 μM) and tested compounds at concentration 10 μM. The following double-stranded 5´-biotinylated oligonucleotides containing DR3 motif from the XREM sequence of CYP3A4 gene promoter were used. Gel mobility shift assay was performed using LightShift Chemiluminescent EMSA Kit (Thermo Scientific, Waltham, MA, USA) as described previously [23,24]. The sequences of DR3 oligonucleotide were: sense 5´-GAATGAACTTGCTGACCCTCT-3´; antisense 5´-AGAGGGTCAGCAAGTTCATTC-3´.

### Statistics

Student´s t-test, One-way ANOVA followed by Dunnett's post test as well as calculations of EC_50_ and IC_50_ values were calculated using GraphPad Prism version 6.0 for Windows, GraphPad Software, La Jolla, California, USA.

## Results

### Cytotoxicity of statin enantiomers in human cancer cell lines

**Fig 2:**
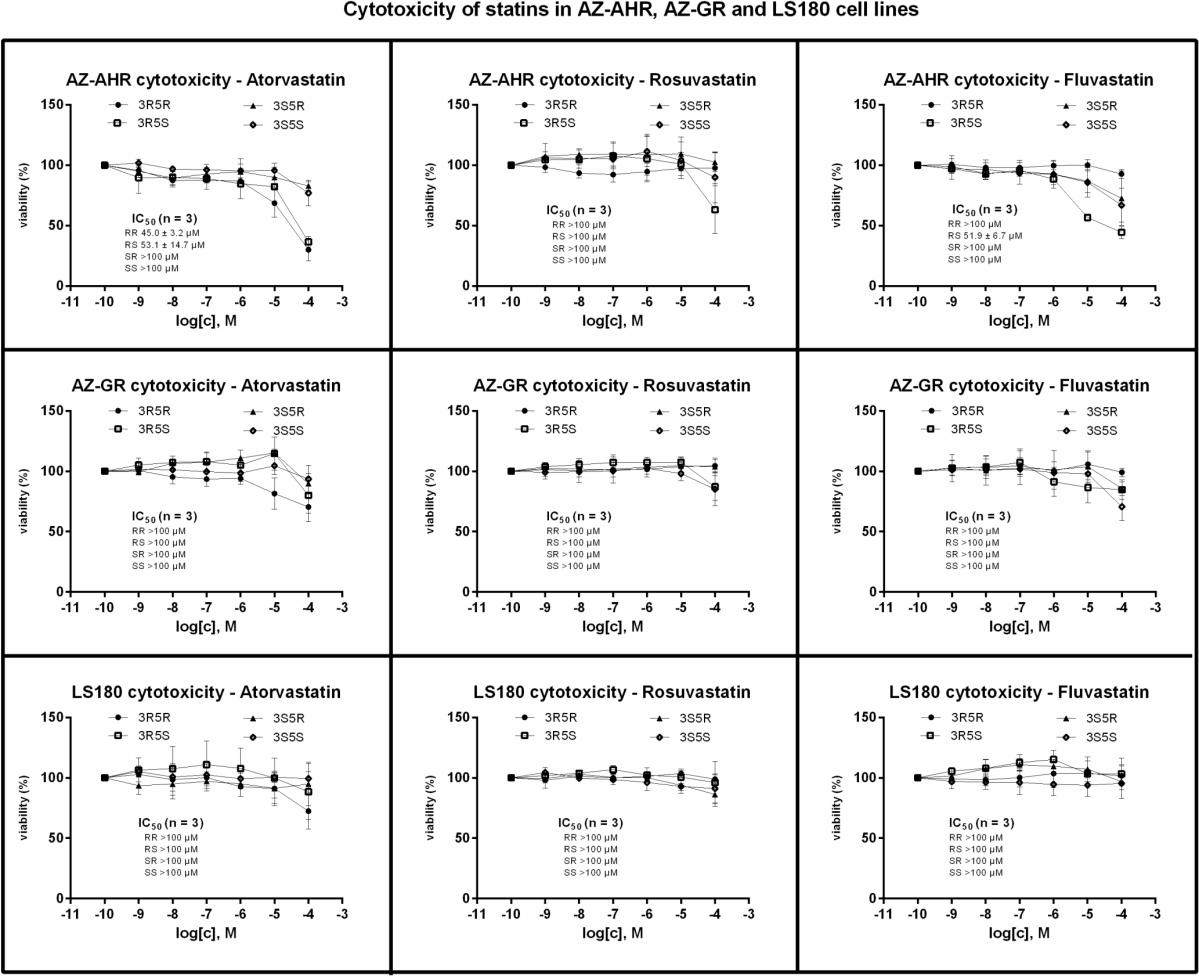
Cytotoxicity of statin enantiomers in human cancer cell lines. AZ-AHR, AZ-GR and LS180 cells were seeded in 96-well plates, stabilized for 16 h and then incubated for 24 h with enantiopure forms of atorvastatin, rosuvastatin and fluvastatin in concentration ranging from 10^−10^ M to 10^−4^ M. The vehicle was dimethylsulfoxide (DMSO; 0.1% v/v). After the treatment, a conventional MTT test was performed and absorbance was measured at 540 nm. Treatments were performed in triplicates. The data are the mean ± SD from experiments performed in three consecutive passages of cells and are expressed as percentage of viability of control cells. The values of IC_50_ were calculated where appropriate and they are indicated in plots. Student´s t-test, One-way ANOVA followed by Dunnett's post test and IC_50_ values were calculated using GraphPad Prism.

Prior to gene reporter assays, we examined the cytotoxicity of tested compounds in AZ-AHR, AZ-GR and LS180 cell lines. For this purpose, the cells were incubated for 24 h with individual enantiomers of atorvastatin, fluvastatin and rosuvastatin at concentrations ranging from 100 pM to 100 μM. The vehicle was DMSO (0.1% v/v). After the treatment, a conventional MTT test was performed and the values of IC_50_ were calculated. Based on the results from cytotoxicity testing (Fig 2), gene reporter assays were performed in concentrations of tested compounds up to 100 μM, with exception of atorvastatin, where maximal concentration of 10 μM was used for incubations in AZ-AHR and AZ-GR cells.

### Effects of statin enantiomers on transcriptional activity of aryl hydrocarbon receptor

**Fig 3:**
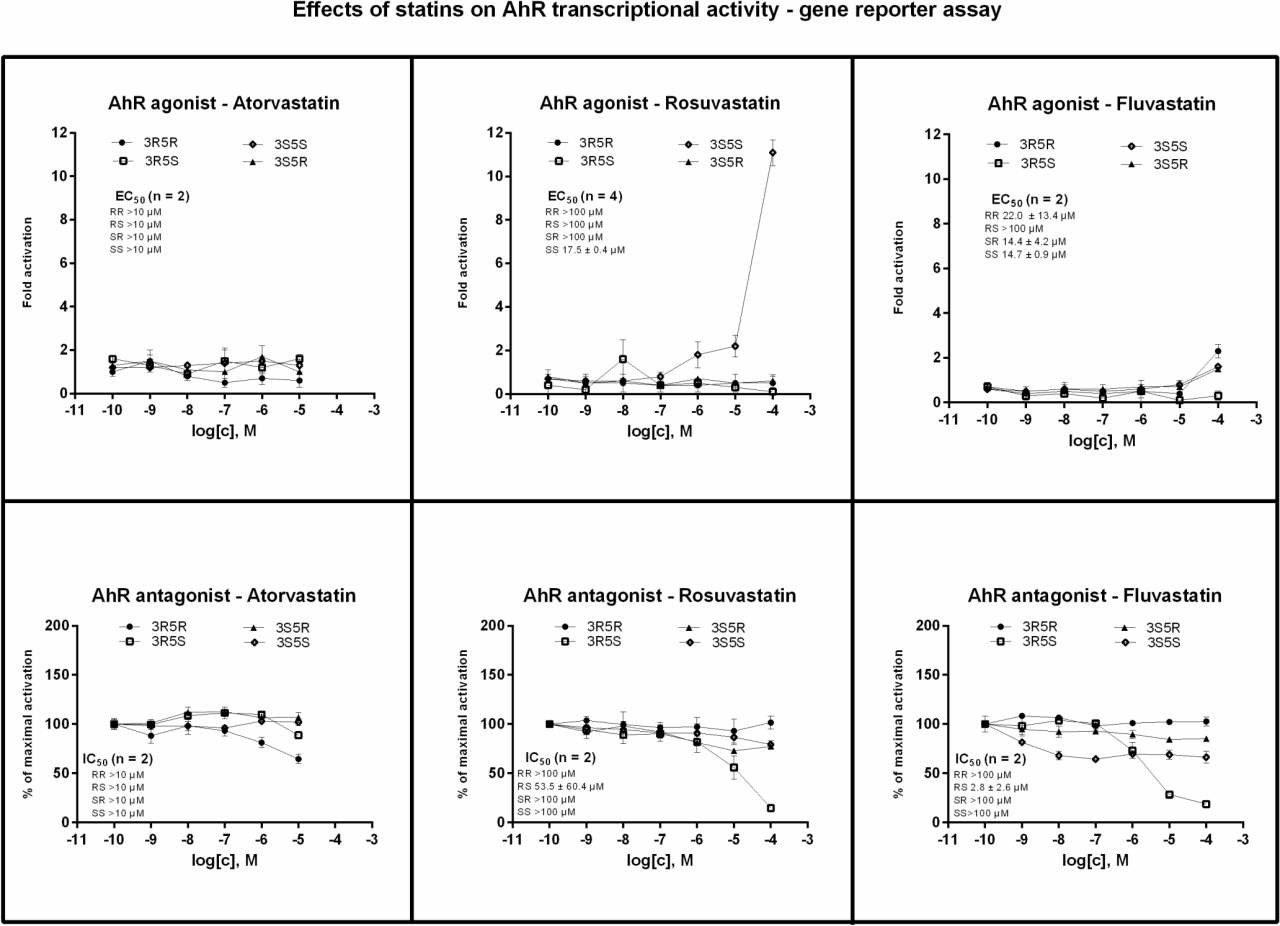
Effects of statin enantiomers on trancriptional activity of human aryl hydrocarbon receptor. AZ-AHR cells were seeded in 96-well plates and stabilized for 16 h and then incubated for 24 h with enantiopure forms of atorvastatin, rosuvastatin and fluvastatin in concentration ranging from 10^−10^ M to 10^−4^ M in the absence (agonist mode–upper panels) or presence (antagonist mode–lower panels) of dioxin (TCDD; 5 nM). The vehicle was DMSO (0.1% v/v). After the treatments, cells were lysed and luciferase activity was measured. Treatments were performed in triplicates. Data are expressed as a fold induction of luciferase activity over control cells (agonist mode) or as a percentage of maximal activation attained by TCDD (antagonist mode). The values of EC_50_ and IC_50_ from *n* independent cell passages were calculated where appropriate and the average values are indicated in plots. Representative gene reporter assays are shown. Student´s t-test, One-way ANOVA followed by Dunnett's post test and EC_50_/IC_50_ values were calculated using GraphPad Prism.

Transcriptional activity of AhR was assessed in human gene reporter cell line AZ-AHR incubated for 24 h with tested compounds. An induction of AhR-dependent luciferase activity by model agonist dioxin (TCDD; 5 nM) in eight consecutive passages of AZ-AHR cells varied from 443-fold to 2353-fold (average induction 1107-fold), as compared to DMSO-treated cells. No significant induction of luciferase activity was observed for any atorvastatin enantiomer. 3S5S-rosuvastatin, but not other optical isomers, dose-dependently increased luciferase activity with average EC_50_ value of 17.5 ± 0.4 μM. 3R5R- fluvastatin, 3S5R- fluvastatin and 3S5S- fluvastatin slightly increased luciferase activity with average EC_50_ values of 22.0 ± 13.4 μM, 14.4 ± 4.2 μM and 14.7 ± 0.9 μM, respectively. However, the efficacy of 3S5S-rosuvastatin was about 0.1% of induction attained by TCDD. Similarly, optical isomers of fluvastatin activated AhR with efficacy about 0.01% (Fig 3). TCDD-inducible transcriptional activity of AhR was dose-dependently inhibited by 3R5S- rosuvastatin and 3R5S-fluvastatin. The decrease of TCDD-inducible luciferase activity correlated with decrease of AZ-AHR cells viability (Fig 2), therefore, the observed effect was rather due to the partial cytotoxicity of the compounds than due to antagonism of AhR. All other forms of rosuvastatin, fluvastatin and all atorvastatin enantiomers did not antagonize AhR. Overall, the gene reporter assays in AZ-AHR cells imply zero clinical or toxicological potential of statin enantiomers in terms of AhR activation.

### Effects of statin enantiomers on transcriptional activity of glucocorticoid receptor

**Fig 4:**
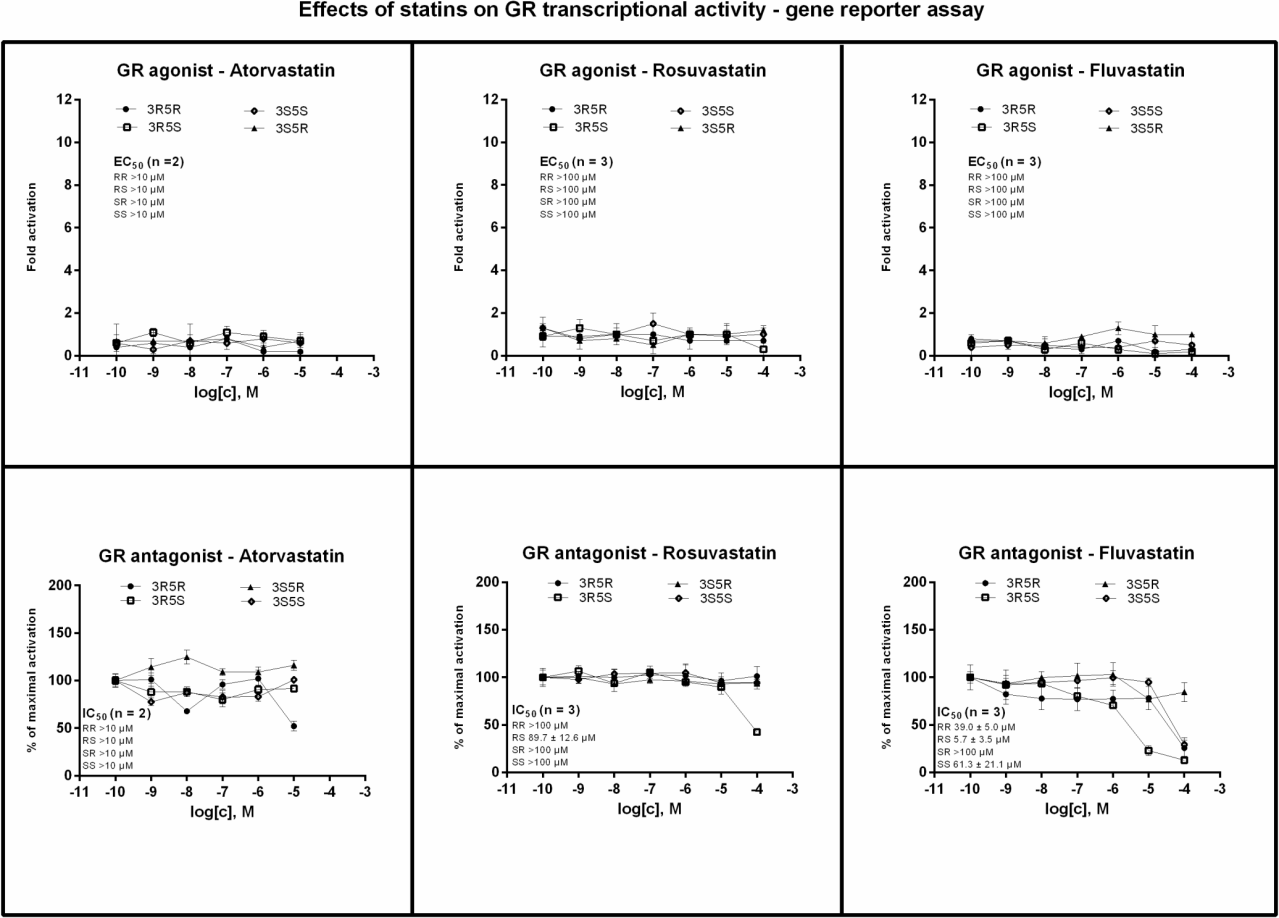
Effects of statin enantiomers on transcriptional activity of human glucocorticoid receptor. AZ-GR cells were seeded in 96-well plates and stabilized for 16 h and then incubated for 24 h with enantiopure forms of atorvastatin, rosuvastatin and fluvastatin in concentration ranging from 10^−10^ M to and 10^−4^ M in the absence (agonist mode–upper panels) or presence (antagonist mode–lower panels) of dexamethasone (DEX; 100 nM). The vehicle was DMSO (0.1% v/v). After the treatments, cells were lysed and luciferase activity was measured. Treatments were performed in triplicates. Data are expressed as a fold induction of luciferase activity over control cells (agonist mode) or as a percentage of maximal activation attained by DEX (antagonist mode). The values of EC_50_ and IC_50_ from *n* independent cell passages were calculated where appropriate and the average values are indicated in plots. Representative gene reporter assays are shown. Student´s t-test, One-way ANOVA followed by Dunnett's post test and EC_50_/IC_50_ values were calculated using GraphPad Prism.

Transcriptional activity of GR was assessed in human gene reporter cell line AZ-GR incubated for 24 h with tested compounds. An induction of GR-dependent luciferase activity by model agonist dexamethasone (DEX; 100 nM) in eight consecutive passages of AZ-GR cells varied from 9-fold to 63-fold (average induction 32-fold), as compared to DMSO-treated cells. None of the tested statins induced GR-dependent luciferase activity. Dexamethasone-inducible transcriptional activity of GR was decreased by 3R5R-atorvastatin (10 μM) and 3R5S-rosuvastatin (100 μM), but the decrease was not dose-dependent and occurred probably due to the cytotoxicity of tested compounds. On the other hand, 3S5S- fluvastatin, 3R5R- fluvastatin, 3R5S- fluvastatin, but not 3S5R- fluvastatin, dose-dependently antagonized GR and there were significant differences between fluvastatin optical isomers (Fig 4).

### Effects of statin enantiomers on transcriptional activity of pregnane X receptor

**Fig 5:**
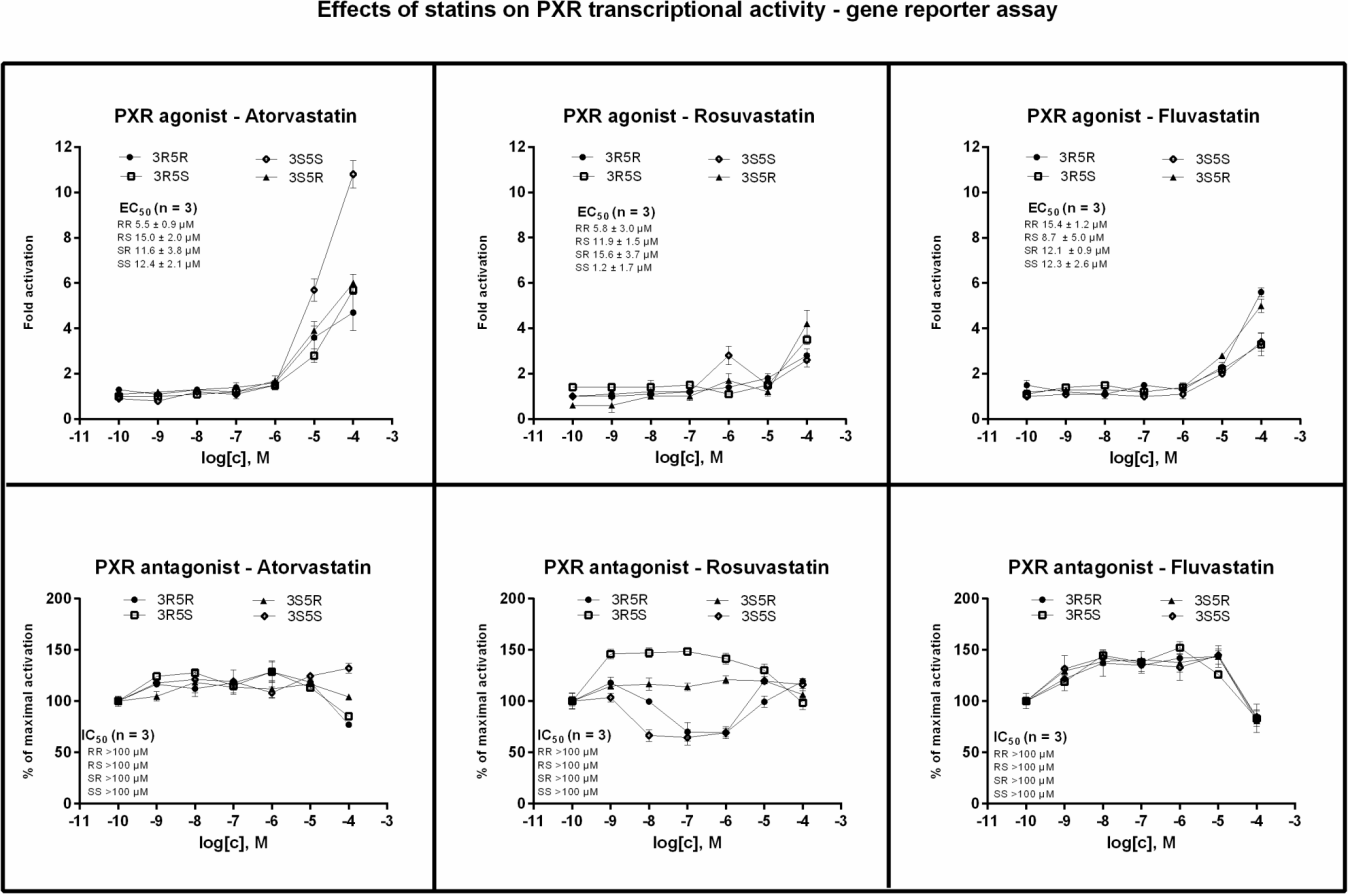
Effects of statin enantiomers on transcriptional activity of human pregnane X receptor. LS180 cells, transiently transfected with p3A4-luc reporter, were seeded in 96-well plates and stabilized for 16 h and then incubated for 24 h with enantiopure forms of atorvastatin, rosuvastatin and fluvastatin in concentration ranging from 10^−10^ M to 10^−4^ M in the absence (agonist mode–upper line) or presence (antagonist mode–lower line) of rifampicin (RIF; 10 μM). The vehicle was DMSO (0.1% v/v). After the treatments, cells were lysed and luciferase activity was measured. Treatments were performed in triplicates. Data are expressed as a fold induction of luciferase activity over control cells (agonist mode) or as a percentage of maximal activation attained by RIF (antagonist mode). The values of EC_50_ and IC_50_ from *n* independent cell passages were calculated where appropriate and the average values are indicated in plots. Representative gene reporter assays are shown. Student´s t-test, One-way ANOVA followed by Dunnett's post test and EC_50_/IC_50_ values were calculated using GraphPad Prism.

Transcriptional activity of PXR was tested in human colon adenocarcinoma cells LS180 transiently transfected with p3A4-luc reporter construct, incubated for 24 h with tested compounds. An induction of PXR-dependent luciferase activity by model agonist rifampicin (RIF; 10 μM) in three consecutive passages varied from 8-fold to 13-fold (average induction 10-fold), as compared to DMSO-treated cells. Transcriptional activity of PXR was dose-dependently induced by all tested statins, and the potency and efficacy between individual optical isomers varied substantially. Ligand-inducible transcriptional activity of PXR was influenced by all tested statins, again differentially, depending on statin and optical isomer:

*FLUVASTATIN*: The potencies of optical isomers of fluvastatin towards PXR were comparable and half maximal effective concentrations EC_50_ ranged from 8.7 μM to 15.4 μM. The efficacies of fluvastatin enantiomers in 100 μM concentrations slightly varied with average inductions of luciferase activity approx. 5-fold (for 3R5R-fluvastatin and 3S5R-fluvastatin) and 3-fold (for 3R5S-fluvastatin and 3S5S-fluvastatin) (Fig 5; upper-right panel). Combined treatments of LS180 cells with PXR agonist rifampicin and enantiomers of fluvastatin revealed inversed U-shaped curves. Dose-dependent augmentation of rifampicin-inducible luciferase activity (150% of rifampicin value) was observed for all statins, regardless optical configuration, in concentrations up to 10^−6^ M, followed by drop of luciferase activity (85% of rifampicin value) at concentrations of statins of 10^−4^ M (Fig 5; lower-right panel). *ROSUVASTATIN*: The efficacies of rosuvastatin enantiomers in 100 μM concentrations were similar and varied around 3-fold induction. The potencies of rosuvastatin optical isomers substantially differed, with following EC_50_ values: 3S5S (1.2 μM) > 3R5R (5.8 μM) > 3R5S (11.9 μM) > 3S5R (15.6 μM) (Fig 5; upper-middle panel). Rifampicin inducible PXR transcriptional activity was not influenced by 3S5R-rosuvastatin, whereas 3R5S-rosuvastatin displayed inverse U-shaped curve, similarly as fluvastatin enantiomers. In contrast, 3R5R-rosuvastatin and 3S5S-rosuvastatin in combination with rifampicin revealed U-shaped curve with minimum at concentrations range 10^−8^ M- 10^−6^ M (60% of rifampicin value) (Fig 5; lower-middle panel). *ATORVASTATIN*: The efficacies of atorvastatin 3R5R- atorvastatin, 3R5S- atorvastatin and 3S5R- atorvastatin in 100 μM concentrations slightly varied with average inductions of luciferase activity approx. 5-fold. Contrary, the efficacy of 3S5S-atorvastatin (100 μM) was much higher, reaching an induction 11-fold. The half maximal effective concentrations EC_50_ for 3S5S-atorvastatin, 3R5S- atorvastatin and 3S5R- atorvastatin ranged from 11.6 μM to 15.0 μM. Interestingly, the potency of clinically used 3R5R-atorvastatin was significantly different from other optical isomers, with EC_50_ of 5.5 μM (Fig 5; upper-left panel). Ligand-inducible transcriptional activity of PXR was not affected by any optical isomer of atorvastatin (Fig 5; lower-left panel). Slight decrease in luciferase activity at 100 μM concentrations of statin corresponds to descrease in cell viability (Fig 2; lower-left panel).

### Effects of statin enantiomers on the expression of drug-metabolizing cytochromes P450 in primary human hepatocytes

We examined a capability of statin enantiomers to induce transcriptionally regulated drug-metabolizing cytochromes P450 in three human hepatocytes cultures (HH59, HH61, HH63). Hepatocytes were treated for 24 h (for determination of mRNA) or 48 h (for determination of proteins) with optical isomers of tested statins (1 μM, 10 μM, 30 μM), dioxin (TCDD; 5 nM), rifampicin (RIF; 10 μM) and vehicle (DMSO; 0.1% v/v).

#### CYP1A1 and CYP1A2

**Fig 6:**
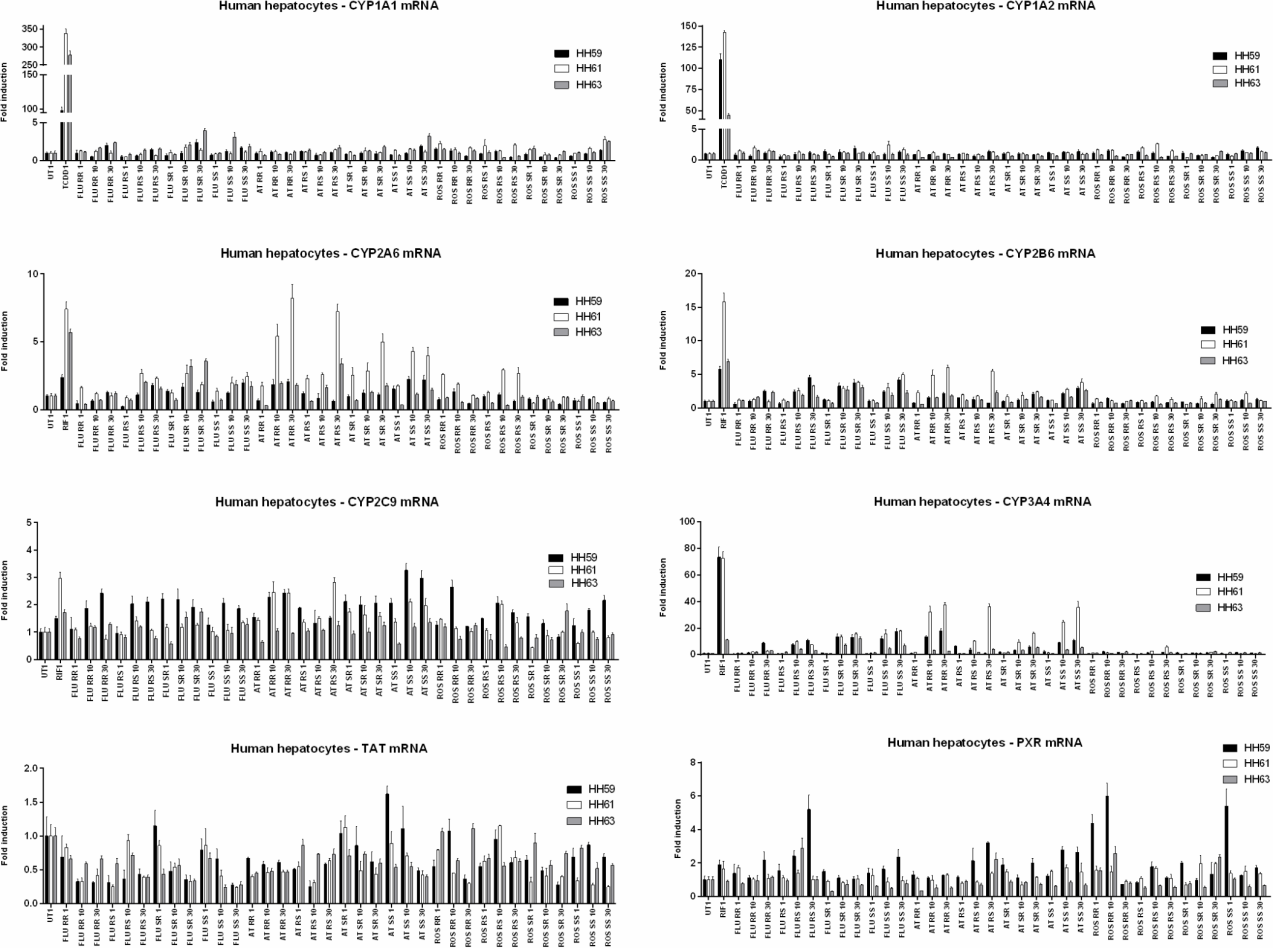
Effects of statin enantiomers on the expression of drug-metabolizing cytochromes P450, PXR and tyrosin aminotransferase TAT at mRNA level in primary human hepatocytes. Primary human hepatocytes from three different donors (HH59, HH61, HH63) were used. Cells were incubated for 24 h with vehicle (DMSO; 0.1% v/v), dioxin (TCDD; 5 nM), rifampicin (RIF; 10 μM) and individual enantiomers of statins (1 μM, 10 μM, 30 μM). Bar graphs of RT-PCR analyses of CYP1A1, CYP1A2, CYP2A6, CYP2B6, CYP2C9, CYP3A4, PXR and TAT mRNAs are shown. The data are the mean ± SD from triplicate measurements and are expressed as a fold induction over vehicle-treated cells. The data were normalized to GAPDH mRNA levels. Student´s t-test and One-way ANOVA followed by Dunnett's post test were calculated using GraphPad Prism.

**Fig 7:**
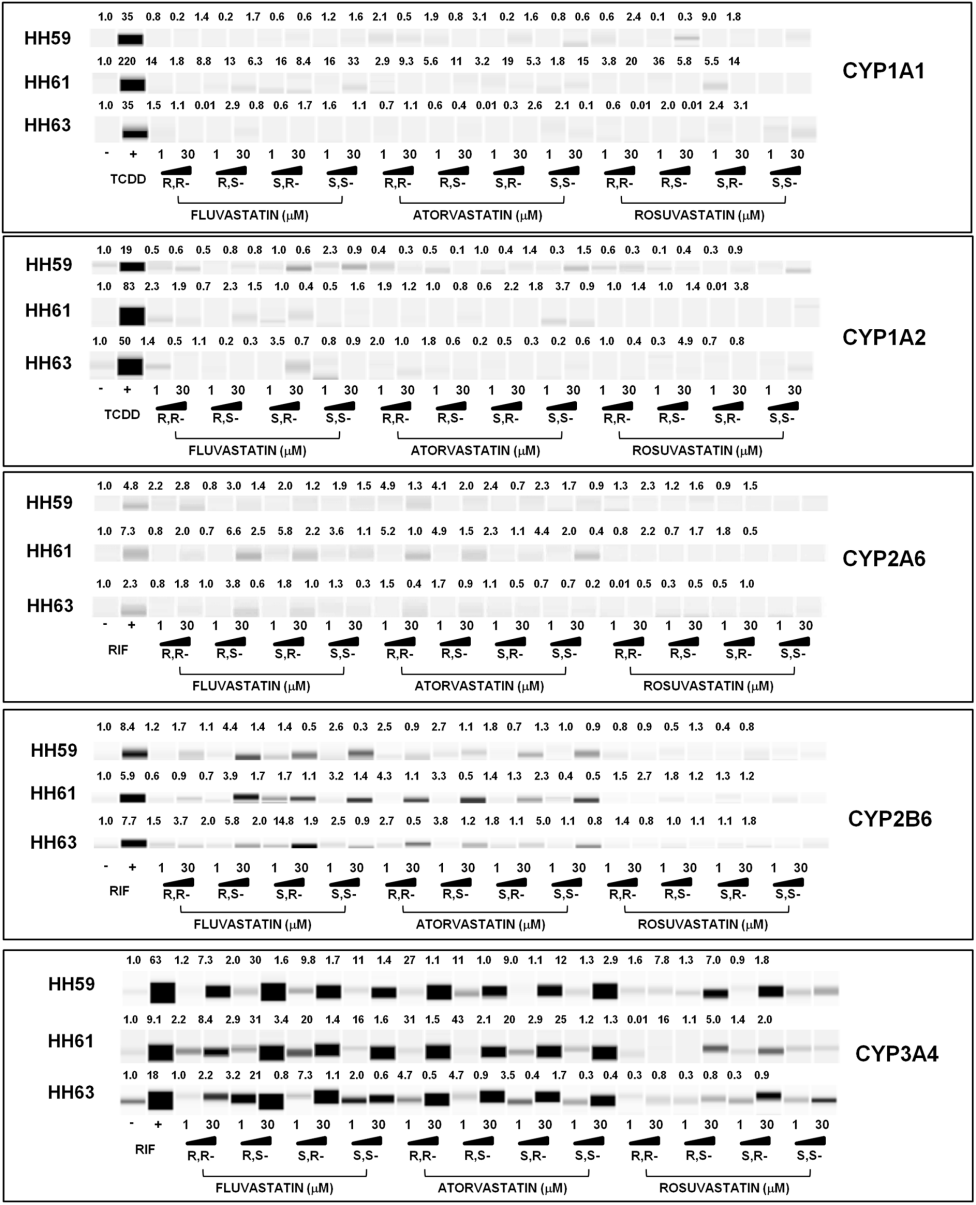
Effects of statin enantiomers on the expression of drug-metabolizing cytochromes P450 at protein level in primary human hepatocytes. Primary human hepatocytes from three different donors (HH59, HH61 and HH63) were used. Cells were incubated for 48 h with vehicle (DMSO; 0.1% v/v), dioxin (TCDD; 5 nM), rifampicin (RIF; 10 μM) and individual enantiomers of statins (1 μM, 30 μM). Simple Western blots of CYP1A1, CYP1A2, CYP2A6, CYP2B6 and CYP3A4 are shown. The data are expressed as a fold induction over vehicle-treated cells and normalized to β-actin levels. *Note*: *analyses of 780 samples are contained in a figure*.

Dioxin strongly induced CYP1A1 and CYP1A2 mRNAs in all human hepatocytes cultures after 24 h of incubation, and the magnitudes of induction in cultures HH59, HH61 and HH63 for CYP1A1/CYP1A2 mRNAs were 98-fold/110-fold, 339-fold/143-fold, and 278-fold/45-fold, respectively. None of the statins tested did significantly induce CYP1A1 or CYP1A2 mRNA in any human hepatocyte culture (Fig 6). Dioxin strongly induced CYP1A1 and CYP1A2 proteins in all human hepatocytes cultures after 48 h of incubation, and the magnitudes of induction in cultures HH59, HH61 and HH63 for CYP1A1/CYP1A2 proteins were 35-fold/19-fold, 220-fold/83-fold, and 35-fold/50-fold, respectively. In line with data at mRNAs level, we did not observe significant induction of CYP1A1 or CYP1A2 protein by any tested statin (Fig 7). Since CYP1A1 and CYP1A2 are dominantly regulated by AhR, the effects of statins on CYP1A1 and CYP1A2 expression are consistent with their effects on AhR in gene reporter assays (Fig 3).

#### CYP2A6

Induction of CYP2A6 mRNA by rifampicin after 24 h of incubation was 2.4-fold, 5.2-fold and 7.1-fold in human hepatocytes cultures HH59, HH61 and HH63, respectively. Induction of CYP2A6 protein by rifampicin after 48 h of incubation was 4.8-fold, 7.3-fold and 2.3-fold in human hepatocytes cultures HH59, HH61 and HH63, respectively. Rosuvastatin enantiomers did not significantly induce the expression of CYP2A6 mRNA and protein, with exception of weak increase of CYP2A6 mRNA in culture HH61by 3R5S-rosuvastatin (10 μM; 30 μM). Induction profiles of CYP2A6 by fluvastatin enantiomers differed among individual cultures, which could be caused by interindivual variability of the donors. Generaly, 3S5S-fluvastatin, 3R5S-fluvastatin, 3S5R-fluvastatin, but not 3R5R-fluvastatin, weakly induced CYP2A6 mRNA and protein. Induction of CYP2A6 mRNA by atorvastatin enantiomers was higher than those by fluvastatin and rosuvastatin in all human hepatocytes cultures, and in some cases even higher that that by rifampicin, implying again interindividual variability between human hepatocytes donors. The magnitude of CYP2A6 induction by atorvastatin enantiomers increased in order: 3R5R > 3R5S = 3S5R > 3S5S (Figs 6 and 7).

#### CYP2B6

Inductions of CYP2B6 mRNA/protein by rifampicin after 24 h/48 h of incubation were 5.8-fold/8.4-fold, 16-fold/5.9-fold and 6.9-fold/7.7-fold in human hepatocytes cultures HH59, HH61 and HH63, respectively. The induction profiles of CYP2B6 by tested statins displayed similar patter as those for CYP2A6. Rosuvastatin did induce neither CYP2B6 mRNA nor CYP2B6 protein in any human hepatocyte culture. We found moderate, dose-dependent induction of CYP2B6 by 3S5S-fluvastatin, 3R5S-fluvastatin, 3S5R-fluvastatin, but not 3R5R-fluvastatin, in all three hepatocytes cultures. Atorvastatin was the strongest inducer of CYP2B6, as compared to rosuvastatin and fluvastatin. Dose-dependent induction of CYP2B6 mRNA and protein by atorvastatin optical isomers increased as follows: 3R5R > 3R5S = 3S5S > 3S5R (Figs 6 and 7).

#### CYP2C9

Induction of CYP2C9 mRNA by rifampicin after 24 h of incubation in three human hepatocytes cultures varied from 1.5-fold to 3-fold. We did not evaluate induction of CYP2C9 protein, because commercial CYP2C9 antibodies were not compatible with SallySue Simple Western System used for analyses. The effects of optical isomers of tested statins on CYP2C9 expression were rather positive modulatory, displaying weak inductions with similar patterns as those for CYP2A6 and CYP2B6 (Figs 6 and 7).

#### CYP3A4

Inductions of CYP3A4 mRNA/protein by rifampicin after 24 h/48 h of incubation were 59-fold/63-fold, 61-fold/9-fold and 11-fold/18-fold in human hepatocytes cultures HH59, HH61 and HH63, respectively. Rosuvastatin did not induce CYP3A4 mRNA, but 3S5R-rosuvastatin and 3R5S-rosuvastatin increased CYP3A4 protein in two human hepatocyte cultures. Fluvastatin induced dose-dependently CYP3A4 mRNA and protein in all human hepatocytes cultures. The effects of 3S5S-fluvastatin, 3R5S-fluvastatin and 3S5R-fluvastatin were nearly equipotent, while 3R5R-fluvastatin was much weaker inducer of CYP3A4 as compared to remaining enantiomers. All optical isomers of atorvastatin strongly and dose-dependently induced CYP3A4 mRNA and protein in all human hepatocytes cultures. The magnitude of CYP3A4 induction differed for individual enantiomers as follows: 3R5R > 3R5S = 3S5S > 3S5R (Figs 6 and 7).

### Effects of statin enantiomers on the expression of PXR and tyrosin aminotransferase TAT mRNAs in primary human hepatocytes

Glucocorticoid receptor GR plays central role in transcriptional regulation of drug-metabolizing enzymes by multiple mechanisms, therefore, we also analyzed the expression of tyrosine aminotransferase TAT (exclusive GR-target gene) and PXR (non-exclusive GR-target gene) in primary human hepatocytes. Primary human hepatocytes are routinely cultured in the presence of dexamethasone in concentration, which fully activates GR. Therefore, the TAT gene is induced under these conditions, and we evaluated either inhibitory or synergistic effects of statins on TAT expression. We observed down-regulation of TAT by some of the compounds tested, however, the effects lacked dose-response pattern and they were not systematic between human hepatocytes cultures. The most frequent and pronounced were the effects by fluvastatin enantiomers (Fig 6), which is consistent with antagonism of GR by these compounds (Fig 4). Similarly, tested statins had mild modulatory effects on the expression of PXR mRNA, however, the effects varied between hepatocytes cultures and were not dose dependent (Fig 6).

### Binding of PXR to DNA—electrophoretic mobility shift assay

**Fig 8:**
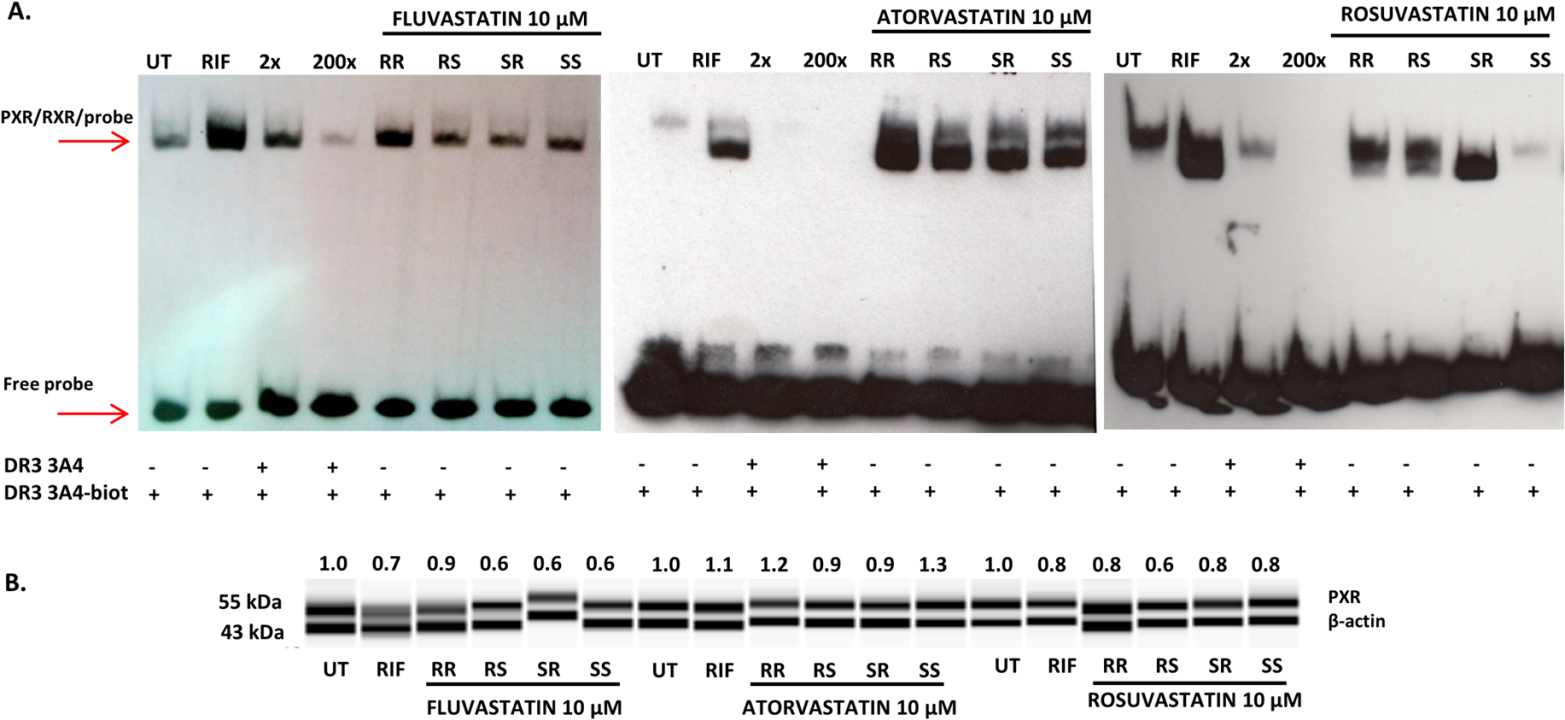
Effect of statin enantiomers on the binding of PXR/RXR complex to the DR3 motif of human CYP3A4 gene promoter. Nuclear fractions of LS174T cells from three independent cell passages were incubated for 2 h at 30°C with DMSO (0.1% v/v), RIF (10 μM) and individual enantiomers of atorvastatin, rosuvastatin and fluvastatin at concentration 10 μM. Treated nuclear extracts were incubated with a biotin-labeled CYP3A4-DR3 probe and electrophoresed on 5% polyacrylamide gel as described under ‘‘Materials and methods section.” **A.** The complex formation of CYP3A4 DR3 response element with PXR-RXRα heterodimer. **B.** Simple western blot showing equal expression levels of PXR nuclear extracts used for EMSA (normalized to β-actin levels).

We tested whether the effects of statins on PXR-CYP3A4 signaling pathway involves also changes in the formation of PXR/RXRα-DNA complex. PXR-mediated gene activation requires direct binding of the PXR-RXRα heterodimeric complex to the response elements in the gene promoter. Maximal induction of CYP3A4 gene expression apparently requires an additional DR3 nuclear receptor-binding element 1(dNR1; -7733/-7719) in a distal xenobiotic responsive enhancer module [25,26]. Nuclear extracts from LS174T cells treated by DMSO (0.1% v/v), RIF (10 μM) and individual enantiomers of atorvastatin, rosuvastatin and fluvastatin at concentration 10 μM were incubated with biotin-labeled double-stranded oligonucleotide corresponding to the DR3 PXR response element in the CYP3A4 promoter and resolved on non-denaturing gel. The specificity of PXR-RXRα binding was confirmed by competition with non-labeled double-stranded DR3 oligonucleotide. Positive control RIF strongly stimulated a formation of PXR/RXRα-DNA-binding complex (Fig 8A). All tested compouds increased binding of PXR/RXRα to the DR3 module in comparison with vehicle. In many samples, the intensity of bands was comparable with that by positive control rifampicin. Given that this method is semi-quantitative, we do not attempt to compare the intensity of individual bands between enantiomers. Immunoblot analysis confirmed that equal amounts of PXR proteins were used in the gel shift assay (Fig 8B).

## Discussion

In the current paper, we investigated enantioselective effects of three, massively used, chiral statins on the expression of inducible drug-metabolizing cytochromes P450 in primary human hepatocytes and on activity of major drug-metabolizing pathways transcriptional regulators. We describe here, for the first time, that atorvastatin, rosuvastatin and fluvastatin enantiospecifically induce CYP2B6, CYP2A6 and CYP3A4 in human hepatocytes, and also that they enantiospecifically influence transcriptional activity of PXR and GR. The discovery of statins was a breakpoint in pharmacotherapy of hypercholesterolemia, therefore, they are also considered as blockbuster drugs. However, numerous drug-drug interactions were reported in patients used statins simultaneously with other drugs. The mechanistic bases for these interactions involve mainly either inhibition or induction of drug-metabolizing cytochromes P450 by statins. The examples of drugs, the metabolism of which is influenced by inhibition of P450 catalytic activity by statins are antihypertensive losartan [27], antidiabetic rapaglinide [28], calcium channel blockers nifedipine [29] and verapamil [30] etc. For instance, fluvastatin was described as a potent inhibitor of CYP2C9 human recombinant enzyme [31], as well as in human liver microsomes and human hepatocytes [32]. Enantiospecific pattern of CYP2C9 inhibition by fluvastatin was described, when K_i_ values were approx. four to five times higher for (-)3S,5R-fluvastatin as compared to (+)3R,5S-fluvastatin [33]. The differences in pharmacokinetic of fluvastatin enantiomers were also observed in humans, when AUC value for (-)3S,5R-fluvastatin was 1.8 times higher than that for (+)3R,5S-fluvastatin [34,35]. Induction of drug-metabolizing CYPs is also a frequent cause for drug-drug interactions. There are numerous reports demonstrating the activation of PXR and induction of PXR-regulated drug-metabolizing cytochromes P450 by statins [10–14].

The statins investigated in the current paper are clinically used as pure enantiomers, i.e. 3R5R-atorvastatin, 3R5S-rosuvastatin and 3R5S-fluvastatin. Since these statins were introduced in a clinical practice directly as enantiopure drugs, the data on induction of drug-metabolizing P450s by optical isomers by tested statins are missing. The rational for use of pure enantiomers is increasing therapeutic efficacy and/or diminishing adverse effects and toxicity of the drug. This concept, unlike in case of statins, led to introduction of enantiopure drugs, which were originally used as racemates. The examples are omeprazole/esomeprazole, citalopram/escitalopram, modafinil/armodafinil, cetirizine/levocetirizin and many others. We have recently described that several clinically used chiral drugs, including ketoconazole [15,16], warfarin [17], omeprazole and lansoprazole [18,19] have enantiospecific effects on PXR, GR and/or AhR and the expression of drug-metabolizing P450s. Taken together, the aim of the current work was to investigate, whether the effects of optical isomers of clinically used chiral statins may induce drug-metabolizing P450s in stereospecific manner, and to compare the effects of clinically used enantiomers with remaining optical isomers of the statins.

None of the tested statins and their optical isomers induced CYP1A1 and CYP1A2 in primary human hepatocytes. Consistently, transcriptional activity of AhR was not influenced by enantiomers of atorvastatin, fluvastatin and rosuvastatin, as revealed by gene reporter assays.

All optical isomers of all tested statins caused formation of PXR-DNA complex in three independent EMSA experiments, but quantitative profiles between enantiomers were not reproducible. Xenobiotics-inducible cytochromes P450, belonging to families CYP2 and CYP3, were differentially induced by all statins. The least active were optical isomers of rosuvastatin, which only moderately activated PXR in gene reporter assays (about 2–4 fold at 100 μM), without significant differences between enantiomers. Rosuvastatin did not induce CYP2/CYP3 genes, with exception of significant induction of CYP3A4 protein, but not CYP3A4 mRNA, by 3R5S-rosuvastatin and 3S5R-rosuvastatin in all human hepatocytes cultures. Enantiomer 3R5S-rosuvastatin also induced CYP2A6 mRNA. Since clinically used optical isomer of rosuvastatin in 3R5S form, the induction of CYP2A6 and CYP3A4 is unfavorable. The induction profiles of CYP2A6, CYP2B6 and CYP3A4 by atorvastatin enantiomers were similar, and the potency of individual optical isomers decreased in order RR>RS = SR>SS. Clinically used atorvastatin is 3R5R form, hence, it is again unfavorable situation with regard to P450 induction-based interactions. Half maximal effective concentration EC_50_ in PXR-gene reporter assays was the lowest for 3R5R-atorvastatin (about 50% of other atorvastatin enantiomers), which is consistent with the fact that CYP2/3 families are dominantly regulated by PXR. On the other hand, the magnitude of luciferase induction was the highest for 3S5S-atorvastatin, which was the weakest inducer of CYP2/3 genes.

Optical isomers of fluvastatin RR, RS and SS displayed dose-dependent antagonistic activity against GR, which is a master regulator of xenobiotic metabolic pathways by multiple mechanisms, including the regulation of PXR expression. Antiglucocorticoid activity of 3R5R-fluvastatin and 3S5S-fluvastatin was also confirmed in human hepatocytes, where we observed down-regulation of TAT mRNA by these two compounds. Therefore, the induction of P450s by fluvastatin comprises both agonist effects on PXR and antagonist effects on GR. The magnitude of induction of CYP2A6, CYP2B6 and CYP3A4 was much weaker by 3R5R-fluvastatin as compared to other three enantiomers.

Besides GR and PXR, important transcriptional regulators of CYP2 and CYP3 genes are nuclear receptors, steroid receptors and also xenoreceptor CAR (Constitutive Androstane Receptor). It was demonstrated that several clinically important statins are activators of FXR (Farnesoid X Receptor) and CAR [11,36]. Indeed, there is mutual cross-talk between PXR and CAR, in terms of sharing response elements, co-activators, target genes and ligands [37]. Therefore, it is likely that tested statins may have enantiospecific effects also against CAR.

A myriad of QSAR (Quantitative Structure-Activity Relationship) studies was performed with clinically used statins both, in the phase of their development and in the research on newly synthesized HMG-CoA inhibitors [38]. On the other hand, no systematic attempts have been made regarding QSAR of stains and their off-targets, e.g. PXR, GR or CAR.

Overall, the current study is the first report on enantioselective effects of statins on the expression of xenobiotic-metabolizing human enzymes. The data contained in the manuscript show that the potential for drug-drug interactions involving induction of P450s is higher for clinically used optical isomers of rosuvastatin, atorvastatin and fluvastatin, as compared to their respective enantiomers, which are not in therapeutic use.
